# Pomegranate Extract Affects Fungal Biofilm Production: Consumption of Phenolic Compounds and Alteration of Fungal Autoinducers Release

**DOI:** 10.3390/ijerph192114146

**Published:** 2022-10-29

**Authors:** Bruna Colombari, Davide Tagliazucchi, Alessandra Odorici, Eva Pericolini, Ismaela Foltran, Diego Pinetti, Aida Meto, Samuele Peppoloni, Elisabetta Blasi

**Affiliations:** 1 Laboratory of Microbiology and Virology, Department of Surgery, Medicine, Dentistry and Morphological Sciences with Interest in Transplant, Oncology and Regenerative Medicine, University of Modena and Reggio Emilia, Via G. Campi 287, 41125 Modena, Italy; 2 Department of Life Sciences, University of Modena and Reggio Emilia, Via Amendola, 2—Pad. Besta, 42100 Reggio Emilia, Italy; 3 Laboratory of Microbiology and Virology, School of Doctorate in Clinical and Experimental Medicine, University of Modena and Reggio Emilia, Via G. Campi 287, 41125 Modena, Italy; 4 Incos-Cosmeceutica Industriale, Funo di Argelato, 40050 Bologna, Italy; 5 Centro Interdipartimentale Grandi Strumenti (C.I.G.S), University of Modena and Reggio Emilia, Via G. Campi 287, 41125 Modena, Italy; 6 Department of Dentistry, Faculty of Dental Sciences, University of Aldent, 1007 Tirana, Albania

**Keywords:** anti-biofilm, *Candida albicans*, in vitro, phenolic compounds, pomegranate, virulence

## Abstract

*Candida albicans* expresses numerous virulence factors that contribute to pathogenesis, including its dimorphic transition and even biofilm formation, through the release of specific quorum sensing molecules, such as the autoinducers (AI) tyrosol and farnesol. In particular, once organized as biofilm, *Candida* cells can elude conventional antifungal therapies and the host’s immune defenses as well. Accordingly, biofilm-associated infections become a major clinical challenge underlining the need of innovative antimicrobial approaches. The aim of this in vitro study was to assess the effects of pomegranate peel extract (PomeGr) on *C. albicans* growth and biofilm formation; in addition, the release of tyrosol and farnesol was investigated. The phenolic profile of PomeGr was assessed by high-performance liquid chromatography coupled to electrospray ionization mass spectrometry (HPLC-ESI-MS) analysis before and after exposure to *C. albicans*. Here, we showed that fungal growth, biofilm formation and AI release were altered by PomeGr treatment. Moreover, the phenolic content of PomeGr was substantially hampered upon exposure to fungal cells; particularly pedunculagin, punicalin, punicalagin, granatin, di-(HHDP-galloyl-hexoside)-pentoside and their isomers as well as ellagic acid–hexoside appeared highly consumed, suggesting their role as bioactive molecules against *Candida*. Overall, these new insights on the anti-*Candida* properties of PomeGr and its potential mechanisms of action may represent a relevant step in the design of novel therapeutic approaches against fungal infections.

## 1. Introduction

The formation of biofilm by numerous microbial species represents an important mechanism of survival and persistence; upon biofilm production, pathogenicity and virulence are also profoundly affected [1,2]. Biofilm formation has been widely recognized as a key step in clinical settings, where biofilm-associated infections occur and commonly affect different anatomical sites, including heart valves, oral cavity or continuity solutions, and medical devices, such as orthopedic prostheses, venous and urological catheters [1].

Biofilm is defined as a complex aggregation of microorganisms embedded in a protective extracellular polymeric substance (EPS), rich also in proteins, lipids and extracellular DNA (eDNA), produced by the microorganism itself. By this extracellular matrix, microbial adhesion to surfaces and consolidation of the biofilm three-dimensional structure are facilitated [2,3]. Increasing studies show that biofilm production increases microbial resistance to environmental stressors, such as temperature, pH and pressure changes, lack of nutrients and antimicrobial agents’ susceptibility as well [4,5,6,7]. Biofilm formation is finely governed and regulated by the quorum sensing (QS), a sophisticated cell-to-cell communication system regulating gene expression and microbial cell behavior in response to fluctuations in cell-population density. QS relies on the release of low molecular weight chemical signaling molecules, called autoinducers (Al) [8], which differ among different microorganisms. 

*Candida albicans* (*C. albicans*) is an opportunistic pathogen, often responsible for biofilm-associated infections, given its ability to tightly and persistently adhere to biotic/abiotic surfaces. Once produced, *Candida* biofilm establishes clinically relevant and difficult-to-treat infections, because of the enhanced resistance of the biofilm-embedded cells to antimicrobial drugs [9]. Four major *C. albicans* AI have been described during biofilm development, namely farnesol, tyrosol, phenylethanol and tryptophol [10,11,12,13]; such molecules are major players not only in fungal morphogenesis (yeast-to-hyphal cell transition) but also in the infectious process, promoting dissemination and establishment of foci in distal anatomical sites [12]. Clinically, this scenario is further complicated by the continuous detection of fungal isolates with newly acquired resistance against conventional drugs, because of mutations and/or genetic recombination [14,15]. Hence, there is a need for identifying novel tools that would exert direct killing and/or anti-biofilm activity, possibly avoiding drug-mediated pressure and consequently developing further antimicrobial resistance [16].

Among natural compounds [17,18], the *Punica granatum* L. peel extract (PomeGr) is an excellent source of a variety of bioactive molecules exerting beneficial health effects, due to their antimicrobial, anti-inflammatory, anticancer and antioxidant properties [19,20,21,22]. In addition, increasing literature ascribes significant antifungal effects to specific compounds, such as phenolic acid, hydrolysed tannins, flavonoids, etc. [23,24]. Recently, we provided initial evidence on the efficacy of PomeGr against biofilm production by *C. albicans* and *Pseudomonas aeruginosa* as well [25].

The aim of the present study was to investigate the anti-*Candida* properties of PomeGr. In particular, we assessed the PomeGr effects on fungal growth, biofilm formation and release of specific auto-inducers, known to be relevant in *Candida* virulence. Furthermore, the consumption of specific phenolic compounds by *Candida* was investigated and their potential role discussed.

## 2. Materials and Methods

### 2.1. Fungal Cells and Growth Condition 

The *C. albicans* engineered strain, endowed with a reporter gene coding for a yeast green fluorescent protein (y-EGFP), was used [26]. In particular, the y-GFP *Candida* cells constitutively emitted a fluorescent signal, measurable by the Fluoroskan reader (Thermo Fisher Scientific, Waltham, MA, USA) at the λ of 490/521 nm; the intensity of the emitted signal, related to the number of viable cells, was quantified and expressed as relative fluorescent units (RFU).

Operationally, fungal cells, maintained at −80 °C in glycerol stocks, were seeded onto Sabouraud Dextrose Agar (SAB) plates (OXOID, Milan, Italy) and incubated overnight at 37 °C. Then, isolated colonies were collected, added to 10 mL of Sabouraud (SAB) broth (OXOID, Milan, Italy) and allowed to grow overnight at 37 °C under gentle shaking. In order to evaluate the fungal concentration, the optical density (OD) was measured at 595 nm (OD_595_) using the Tecan Sunrise (Tecan Group Ltd., Männedorf, Switzerland) spectrophotometer. 

### 2.2. Pomegranate Peel Extract 

The *Punica granatum* L. peel extract (PomeGr), supplied by INCOS COSMECEUTICA INDUSTRIALE (Bologna, Italy) and produced by PHENBIOX SRL (Bologna, Italy), contained the peel extract (22.5% *w*/*w*), Saccharomyces ferment lysate filtrate, citric acid, sodium benzoate and potassium sorbate. Further details are given as supplementary material (Table S1). The same solution containing all the ingredients, but not PomeGr, was employed as negative control (neg-C). The PomeGr and the neg-C were filtered through a 0.22 µm membrane and maintained at 4 °C, prior for testing in the assays described below.

### 2.3. Fungal Growth and Biofilm Formation Assays

The PomeGr and the neg-C were serially diluted (from 1:2 to 1:128) and plated (100 μL/well) in black 96 well-microtiter plates with transparent bottom; then, the fungal cell suspension (10^6^ cells/mL in SAB obtained from overnight cultures) was added (100 μL/well) and the plates were incubated at 37 °C for 24 h. After incubation, fungal growth (direct reading at 24 h) and biofilm formation (reading after 2× washing of the wells with buffered saline to remove the planktonic cells) were assessed. By the Fluoroskan (Thermo Fisher), the fluorescence signal was measured and the results were expressed as relative fluorescence units (RFU), using previously described protocols with minor modifications [27]. By the Tecan Sunrise, the reading at two different ODs, namely OD_595_ (for microbial growth analysis) and OD_540_ (after crystal violet staining for Biofilm determination), were performed, according to standard procedures.

Moreover, calibration curves were generated employing the colony forming units (CFU) assay. Briefly, serial dilutions of fungal cells were prepared in SAB broth, seeded in black 96 well-microtiter plates with transparent bottoms (100 μL/well) and incubated for 24–48 h at 37 °C. Then, the microtiter plates were read at the Fluoroskan to evaluate the RFU and at the Tecan Sunrise to measure the OD. In parallel, from each well, the CFU assay was performed to quantify the viable cells. Finally, standard curves were generated which allowed the expression of the OD or RFU values in terms of CFU/mL. 

### 2.4. Mass Spectrometry Analysis of PomeGr Extract

High-performance liquid chromatography-electro spray high resolution-mass spectrometry (HPLC-ESI/HR-MS) analysis was performed using an Ultimate 3000 UHPLC coupled to a QExactive Mass Spectrometer via a HESI-II electrospray ion source (Thermo Scientific). Target compounds from pomegranate extracts were detected in 10 μL sample volume injected onto a Hypersil Gold C18 100 × 2.1 mm ID 1.9 μm ps column (Thermo Scientific) kept at 30 °C. Chromatographic separation was performed at 0.5 mL/min flow with a gradient elution scheme using 0.1% formic acid in acetonitrile (B) and 0.1% formic acid in water (A). The mobile phase composition was kept at 2% B for 0.5 min after injection, then linearly raised to 18% B in 27 min and further on to 98% B in 4.5 min. Solvent B was kept at 98% up to minute 34.9 then lowered to 2% at minute 35. The total runtime was 45 min. The HESI-II source was operated in alternating positive and negative ionization mode. Full MS (70,000 FWHM resolution power, 160–800 Th range) with data-dependent MS2 (17,500 FWHM, Top 4 precursor ions) spectra acquisition was performed in both polarities. Analysis was carried out on cell free supernatants from microbial cultures (10^6^/mL) eventually exposed to diluted (1:16) pomegranate extracts for 24 h at 37 °C. The supernatants were transferred to Amicon-Ultra 0.5 tubes, centrifuged at 14,000 rpm for 15 min, diluted 1:1 (vol/vol) with 5% methanol in water and transferred to the autosampler. Using the Thermo Fisher FreeStyle program the relative amount of each compound was calculated by integrating the area under the peak (AUP). AUP was measured from the extracted ion chromatograms (EIC) obtained for each compound with a tolerance ±5 ppm.

### 2.5. Mass Spectrometry Analysis of Fungal Autoinducers 

Cell-free supernatants from *Candida,* treated or not with PomeGr, were analyzed by HPLC-ESI-MS, as detailed elsewhere [17,27,28]. In particular, 24 h–old supernatants from *Candida* cells (10^6^/mL) that had or had not been exposed to PomeGr (1:16 dilution) were harvested, filtered and assessed by MS. The relative amount of the identified autoinducers (AI) was determined as reported in the previous paragraph.

### 2.6. Statistical Analysis

**Figure 1:**
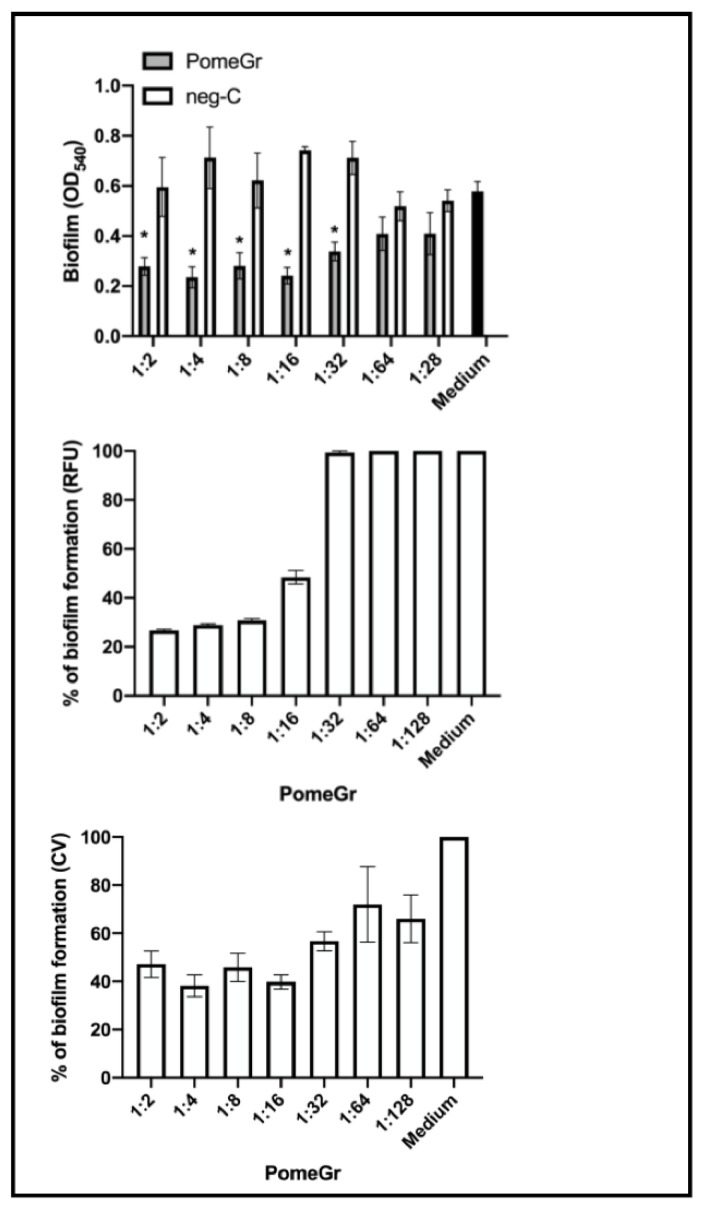
Antibiofilm activity of PomeGr. *C. albicans* (10^6^/mL) was exposed to PomeGr or neg-C at the indicated dilutions, and incubated at 37 °C for 24 h; then, after removing planktonic cells, biofilm was quantified. Upper panel: 24 h-biofilm formation by *Candida* exposed to Pomegr (grey bars) or negative control (white bars), assessed by CV assay. The black bar represents the biofilm production by *C. albicans* in medium only. Central panel: percentage of biofilm formation by *C. albicans* exposed to PomeGr, evaluated by fluorescence assay. Lower panel: percentage of biofilm formation by *C.* *albicans* exposed to PomeGr, as evaluated by CV assay. The data are the means ± SEM of nine replicates of three independent experiments. The asterisks indicate statistically significant differences: * *p* < 0.05 (PomeGr-treated vs. neg-C samples).

The statistical analyses were performed by using GraphPad Prism 8 software. Firstly, the Shapiro–Wilk test was used to analyze the distribution of the data within each experimental group. Differences between groups were analyzed by the Kruskal–Wallis’s test, followed by Dunn’s multiple comparisons test for Table 1 data; while, for Figure 1 data, statistical differences between groups were analyzed according to one-way ANOVA followed by Sidak’s multiple comparisons tests. Values of *p* ≤ 0.05 were considered statistically significant. 

## 3. Results

### 3.1. Anti-Candida Activity of PomeGr 

Initially, the effect of PomeGr was evaluated on total fungal load, following 24 h *Candida* exposure to different dilutions and subsequent evaluation of the RFU. As shown in Table 1 (upper panel), PomeGr affected *Candida* in a dilution-dependent manner; in particular, a significant decrease (*p* < 0.05) in the RFU occurred at dilutions between 1:2 and 1:16, indicating a reduction above 70%. 

To strengthen these data, further experiments were performed by a different assay, namely assessing the fungal load at 24 h, in terms of OD_595_ in treated and untreated cells. In line with the fluorescence results, as shown in Table 1 (lower panel), the levels of OD_595_ varied according to the PomeGr dilution used, with the 1:32 condition still causing significant inhibitory effects. When parallel groups were exposed to the neg-C, no differences in OD_595_ were observed with respect to *Candida* incubated with medium only. Thus, by a different assay, we confirmed the anti-*Candida* activity of the PomeGr and ruled out any potential interference by additive or preservatives included in the formulation. 

Then, we investigated the ability of PomeGr to affect *Candida* biofilm production. In particular, *C. albicans* was incubated under conditions that allowed biofilm production in the presence of PomeGr or neg-C. After 24 h, the wells were washed to the remove planktonic cells; then, the amounts of biofilm produced was measured by fluorescence reading and OD_540_ reading after crystal violet staining. The RFU results, depicted in Figure 1 (upper panel), showed that PomeGr affected biofilm production in a dilution-dependent manner, while the neg-C had no effects. When these same data were expressed as percent biofilm formation, established by either considering the OD_540_ (central panel) or the RFU (lower panel), a relevant impairment was consistently observed till 1:16 (as assessed by fluorescence assay) and 1:32 dilutions (as established by OD measurement after crystal violet staining).

Differently, at dilutions ≥ 1:32, PomeGr had no effects on *Candida* biofilm formation, being the raw OD and RFU values comparable to those observed with the untreated control cells (Medium). As 1:16 was the lowest PomeGr dilution that significantly impaired both *Candida* growth and biofilm production, we focused on this condition for further analyses. According to the literature, this condition also allowed to use the preservatives (potassium sorbate, sodium benzoate and citric acid) at working conditions below the minimal inhibitory concentrations (MIC) previously reported for candida [29,30,31]. 

### 3.2. Biochemical Profile of Pomegr Exposed or Not to Candida

**Figure 2:**
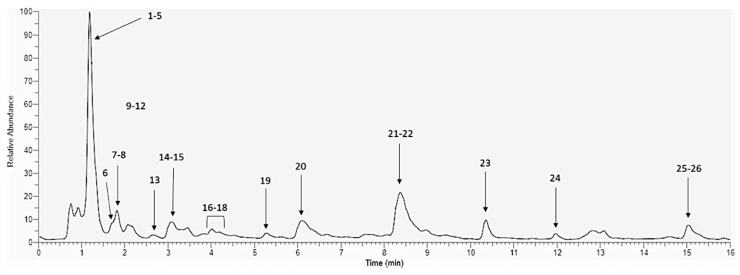
Polyphenols profile of PomeGr. The PomeGr was analyzed by HPLC-ESI-MS. A negative ion mode total ion chromatogram (TIC) is shown, as representative of three independent experiments. The code number of each peak and the corresponding molecules are further detailed in the supplementary material (Table S3).

PomeGr extract is known to be a complex mixture of components, such as polyphenols with a broad spectrum of activities, including antimicrobial, antioxidant and anti-inflammatory effects [31,32,33,34,35]. Initially, to establish the biochemical profile of the PomeGr extract used in this study, HPLC-ESI-MS analysis was performed and a representative chromatogram is shown in Figure 2. 

Details on the mass spectral data of the compounds detected in our extract are given in Table S2. By comparing the MS and MS^2^ profile with literature, several compounds were identified, as detailed in Table S3. Taken together, our data underline the variety of the PomeGr in terms of polyphenols content, similarly to what has been described in the literature [21].

Next, we assessed the possible changes in PomeGr polyphenol content upon exposure to Candida. Thus, PomeGr was incubated for 24 h with and without Candida (10^6^/mL); then, cell-free supernatants were subjected to HPLC-ESI-MS analysis. By chromatogram analysis, the different elution peaks and their areas were used for semi-quantitative evaluation of the specific products, detectable in treated and untreated cells.

**Figure 3:**
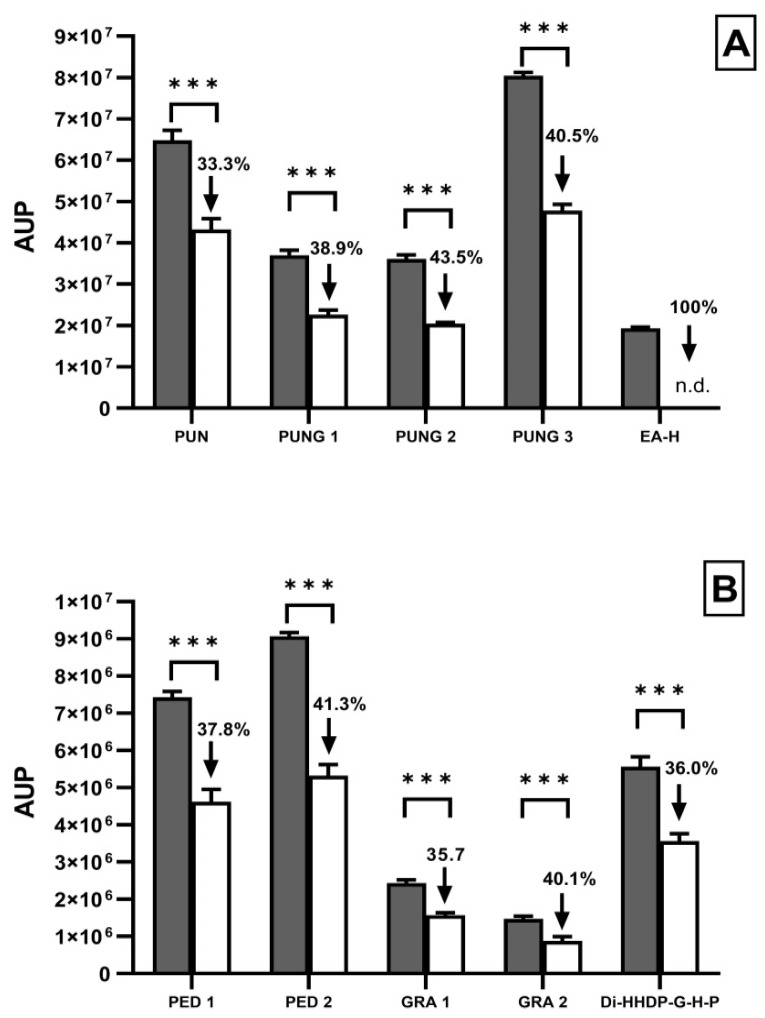
Changes in ellagitannins concentration in PomeGr exposed or not to *C. albicans.* Phenolic compounds were identified by HPLC-ESI-MS in PomeGr alone and after exposure to *C. albicans* (24 h). The relative amounts of each polyphenol were measured by the ion chromatogram, according to the peak area of each compound, and were expressed as area under the peak (AUP) arbitrary units (tolerance ± 5 ppm). Dark grey bars: PomeGr alone; white bars: PomeGr exposed to *C. albicans*. The % decrease (numbers above the columns) was calculated comparing the AUP of treated vs untreated groups. n.d.: compound not detected. The various compounds are shown in panel (**A**,**B**). *** *p* < 0.005. Abbreviations: PUN, punicalin; PUNG 1, punicalagin isomer 1; PUNG 2, punicalagin isomer 2; PUNG 3, punicalagin isomer 3; EA-H, ellagic acid-hexoside; PED 1, pedunculagin 1; PED 2, pedunculagin 2; GRA 1, granatin 1; GRA 2, granatin 2; Di-HHDP-G-H-P, Di-(HHDP-galloyl-hexoside)-pentoside.

Figure 3 shows the peak areas (AUP) of selected phenolic compounds, that had been detected in the PomeGr (grey columns) and were affected upon exposure to *C. albicans* (white columns). In addition, the percent variations of each compound were calculated, as ratio between the AUP in presence and absence of the fungal cells. As detailed by the columns, a reduction in the peak areas of pedunculagin, punicalin, punicalagin, granatin, Di-(HHDP-galloyl-hexoside)-pentoside and their isomers was observed upon PomeGr exposure to *C. albicans*, the decrease ranging between 33.3 and 43.5%, while a drop to undetectable levels was observed for ellagic acid–hexoside; all the other compounds showed low-to-partial reductions (from 3.8 to 21.0% decrease).

### 3.3. Alteration in the Release of AI by Candida upon Treatment with PomeGr 

Finally, we assessed the effects of PomeGr on the ability of C. albicans to produce specific AI, particularly tyrosol and farnesol, known to be involved in fungal biofilm formation and virulence [32]. Thus, HPLC-ESI-MS analysis was performed in cell-free supernatants from *C. albicans*, treated or not with PomeGr for 24 h at 37 °C. By chromatogram analysis, the different elution peaks and their areas were used for semi-quantitative evaluation of the specific products detected in treated and untreated cells. Table 2 shows that untreated *Candida* cells secreted consistent amounts of tyrosol, in line with our recently published data [32,33]. 

The treatment with PomeGr affected such secretion, producing a decrease of 60.9%. Furthermore, in contrast to what observed testing tyrosol, farnesol resulted undetectable in control *Candida* cells but was consistently induced upon treatment, implying an opposed regulation of these two AI by PomeGr.

## 4. Discussion

Here, we show that PomeGr extract impairs *Candida*. Fungal growth and biofilm formation are inhibited and also the release of specific auto-inducers happens to be affected.

*C. albicans* is an opportunistic pathogen, responsible for mild to severe and even life-threatening diseases. Notoriously, it can shift its role from commensal to pathogen, undergoing yeast-to-hyphal form transition and producing biofilm; by doing this, *Candida* can easily evade a host’s immune defenses and even resist to exogenous insults, such as disinfectants and antifungal agents [4,5,6,7].

Kupnik et al. described the antimicrobial activity of plant extracts, including *Punica granatum* L. [34]. Recently, we have provided initial evidence on the ability of PomeGr to affect biofilm formation by both fungal and bacterial cells [25]. To better understand the phenomenon, here, we demonstrate that PomeGr exerts anti-*Candida* activity in a dose-dependent manner. In particular, the 1:16 dilution significantly impairs both fungal load and biofilm formation, by 71.4% and 51.3%, respectively. Such PomeGr-mediated inhibition never reaches 80%, in line with other data [35], documenting the consistent but always partial efficacy of this natural product. Nevertheless, its success may be strikingly enhanced by a combination with conventional drugs; accordingly, it has been demonstrated that punicalagin, a component of PomeGr extract, synergistically interacts with fluconazole against *C. albicans* and *C. parapsilosis* [36]. Similarly, by an in vitro study aimed at identifying novel options to counteract oral infections [35], we have shown that PomeGr, used in combination with a new biomimetic compound that favors enamel remineralization, exerts additive effects against *Candida* biofilm production. Thus, because of their intrinsic properties, PomeGr as well as other natural compounds endowed with antimicrobial activities deserve special interest, not only for the expected additive/synergistic effects but also because they are expected not to induce resistance, as opposed to conventional drugs. 

As known [37,38], pomegranate is an excellent source of bio-compounds with beneficial effects on human health. The relative quantities and compositions of such bioactive molecules may depend on the type of cultivar, the part of the fruit used and the ripening stage [39]. As determined by HPLC-ESI-MS analysis, the PomeGr used in the present study is mainly composed of pedunculagins and their isomers, ellagic acid hexoside, punicalagins and their isomers, punicalin, granatin and Di-(HHDP-galloyl-hexoside)-pentoside; in addition, other polyphenols are also present in low quantities (Figure 2). The obtained profile closely recalls what has been reported for other PomeGr extracts [21], thus underlying that the polyphenolic components actually remain the most abundant components. Some of them have been characterized for antibacterial and antifungal properties [24]; yet, their mechanism(s) of action remains still poorly understood. Here, we demonstrate the consumption of some PomeGr bioactive molecules upon exposure to *Candida*, envisaging their possible role as antibiofilm compounds. In particular, the comparison between the peak areas (AUP) obtained from *Candida* exposed or not to PomeGr (24 h of incubation, to allow biofilm formation/impairment) allows us to establish a decrease in pedunculagin, granatin, Di-(HHDP-galloyl-hexoside)-pentoside and punicalagin content, ranging from 35.7% to 41.3%. Interestingly, the ellagic acid-exoside undergoes complete consumption (it drops to undetectable levels). Paralleling with the biofilm reduction, the decreases observed by HPLC-ESI-MS analysis suggest that pedunculagin, granatin, Di-(HHDP-galloyl-hexoside)-pentoside, punicalagin and ellagic acid-hexoside are indeed molecules actively involved in the accomplishment of the antibiofilm activity. Similarly, the total consumption of ellagic acid, observed in our model, implies its direct role against *Candida*. In agreement with our data, Nejatbakhsh et al. [40] have shown that ellagic acid impairs both growth and biofilm formation by *C. albicans*; in addition, the expression of two genes, hypha-specific wall protein 1 (HWP1) and agglutinin-like sequence 3 (ALS3), known to be involved in adhesion and dimorphyic transition, happens to be deeply suppressed. Taken together, those of [40] and present findings strengthen the relevance of fungal adhesion and filamentation in the establishment of a sessile community and underline the impact of PomeGr and its compounds as potentially useful antibiofilm agent.

Biofilm development is closely regulated by QS, a cell-to-cell communication system that in turn modifies gene expression through the release of specific molecular signals, the AI. Accordingly, *C. albicans* produces several AI, including farnesol and tyrosol. The former is involved in the inhibition of filamentation and subsequent impairment in biofilm formation [41,42,43,44,45]; conversely tyrosol, shortens the fungal lag phase during growth and accelerates germ tube formation, thus promoting the yeast-to-hyphal cell transition [39]. Here, we show that *Candida* treatment with PomeGr impairs tyrosol production by about 60%. In contrast, farnesol, undetectable in untreated samples, is abundantly produced upon PomeGr treatment. Thus, by up-regulating farnesol (a filamentation inhibitor) and down-regulating tyrosol (a filamentation promoter), PomeGr interferes with *Candida* biofilm formation, at least under the conditions employed in our model. Certainly, the opposed effects observed on farnesol and tyrosol warrant further investigations; in any case, we may conclude that the regulation of these two AI is a key step in the accomplishment of antibiofilm effects by PomeGr. It should be noted that the present data are in line with recent papers demonstrating the complex modulation of *Candida* tyrosol/farnesol by other bioactive compounds [32,33]. 

Although having the limitations intrinsic of an *in vitro* model, our data adds new insights on the events occurring during *Candida* biofilm formation and its impairment upon treatment with the natural compound PomeGr. Any potential role of the preservatives may be excluded, being such compounds used at doses below their anti-*Candida* MIC. A better understanding of the events involved in such a phenomenon may help to counteract *Candida* pathogenicity, hopefully targeting selected/novel traits of its virulence. Preliminary experiments rule out a direct toxic effect of PomeGr against human epithelial cells; indeed, when used at 1:16 dilution, it did not affect LDH release (data not shown), further encouraging studies aimed at characterizing novel antimicrobial formulations, possibly retaining a wide-spectrum activity. 

## 5. Conclusions

Our results reinforce the importance of PomeGr as a natural product against the opportunistic pathogen *C. albicans*. Fungal load and biofilm formation are indeed affected in parallel with the consumption of certain phenolic compounds. Furthermore, we provide the first evidence that *Candida* exposure to PomeGr results in opposed regulation of the two major AI, farnesol and tyrosol. Since the finely AI-regulated biofilm formation is linked to *C. albicans* pathogenicity, PomeGr should be considered as a precious source of natural bioactive compounds for the development of new therapeutic strategies against *Candida* infections. 

## Figures and Tables

**Table 1:** Anti-*Candida* activity of PomeGr.

Dilution	RFU ± SEM	% Decrease	CFU/mL ± SEM	% Decrease
**1:2**	0.45 ± 0.027 *	77.2	2.4 × 10^6^ ± 1.8 × 10^5^	94
**1:4**	0.49 ± 0.007 *	75.4	3.4 × 10^6^ ± 3.0 × 10^5^	92
**1:8**	0.51 ± 0.013 *	74.3	4.0 × 10^6^ ± 3.2 × 10^5^	90
**1:16**	0.57 ± 0.020 *	71.4	5.4 × 10^6^ ± 5.0 × 10^5^	87
**1:32**	1.15 ± 0.082	42.2	2.0 × 10^7^ ± 2.0 × 10^6^	51
**1:64**	1.42 ± 0.016	28.7	2.7 × 10^7^ ± 3.3 × 10^6^	35
**1:128**	2.19 ± 0.066	0	4.6 × 10^7^ ± 5.2 × 10^6^	0
**Medium**	1.995 ± 0.025	-	4.1 × 10^7^ ± 6.8 × 10^5^	-
**Dilution**	**Levels of OD_595_ ± SEM**		**CFU/mL × 10^6^ ± SEM**	
	**PomeGr**	**neg-C**	**PomeGr**	**neg-C**
**1:2**	0.21 ± 0.021 *	1.17 ± 0.084	13.06 ± 2.14	109.06 ± 8.39
**1:4**	0.20 ± 0.022 *	1.18 ± 0.084	12.38 ± 2.24	110.88 ± 8.36
**1:8**	0.19 ± 0.022 *	1.19 ± 0.088	11.36 ± 2.19	111.46 ± 8.80
**1:16**	0.23 ± 0.037 *	1.20 ± 0.091	15.22 ± 3.68	112.39 ± 9.13
**1:32**	0.68 ± 0.072 *	1.20 ± 0.082	60.37 ± 7.23	112.43 ± 8.25
**1:64**	0.87 ± 0.060	1.07 ± 0.056	79.18 ± 6.02	99.05 ± 5.58
**1:128**	0.97 ± 0.056	1.11 ± 0.086	89.56 ± 5.63	103.79 ± 8.60
**Medium**	0.94 ± 0.02		86.44 ± 2.05	

*C. albicans* (10^6^/mL) was exposed to PomeGr at the indicated dilutions and incubated at 37 °C for 24 h; then, the fluorescent signal (RFU) or the OD_595_ were measured by Fluoroskan or Tecan reader, respectively. Upper panel: The RFU values of cells exposed to the various PomeGr dilutions and the percent decrease (with respect to cells incubated in medium only) are shown. Lower panel: the OD_595_ levels of PomeGr-treated and neg-C groups are shown. By the RFU and OD calibration curves, the predicted CFU/mL were calculated and shown in the right part of each panel. The data are the mean ± SEM of eleven replicates from two independent experiments. The asterisks indicate statistically significant differences (medium vs. treated samples): * *p* < 0.05.

**Table 2:** AI release by *C. albicans* exposed or not to PomeGr.

Levels of AI				
Treatment	Tyrosol	% Decrease	Farnesol	% Decrease
	(AUP)		(AUP)	
Medium	8.08 × 10^7^	-	n.d.	-
PomeGr	3.16 × 10^7^	60.89	2.15 × 10^7^	n.m.

Cell-free supernatants from *C. albicans*, treated or not with PomeGr, were tested by HPLC-ESI-MS analysis. The indicated AI were investigated. By chromatogram analysis, the different elution peaks were identified, and the relative peak areas (AUP) were used for semi-quantitative evaluation of each specific product. The % decrease was calculated comparing the AUP of treated and untreated groups. The results shown are from a pool of four replicates of a representative experiment out of two performed. n.d.: not detectable; n.m.: not measurable.

## Data Availability

The data presented in this study are available on request from the corresponding authors.

## Supplementary Materials

The following supporting information can be downloaded at: https://www.mdpi.com/article/10.3390/ijerph192114146/s1, Table S1: Mass spectral data of the phenolic compounds identified in the pomegranate extract. Table S2: Mass spectral data of the identified compounds. Table S3: Polyphenolic content in PomeGr exposed or not to *C. albicans*.
