# Supplementary material for: Pomegranate Extract Affects Fungal Biofilm Production: Consumption of Phenolic Compounds and Alteration of Fungal Autoinducers Release
Source: Int J Environ Res Public Health. 2022 Oct 29;19(21):14146. doi: 10.3390/ijerph192114146 (PMC9657062; doi:10.3390/ijerph192114146)
Supplement: Supplementary file 1 [file ijerph-19-14146-s001.zip › ijerph-1863977-supplementary.pdf]

Table S1. Composition of Pomegranate peel extract.

INCI name	% w/w	CAS	EINECS
AQUA	Q.S. to/Q.B. a 100	7732-18-5	231-791-2
PUNICA GRANATUM PEEL EXTRACT	22.5	84961-57-9	284-646-0
SACCHAROMYCES FERMENT LYSATE FILTRATE	1-4	-----------	------------
CITRIC ACID	1.5	77-92-9 / 5949-29-1	201-069-1
SODIUM BENZOATE	0.2	532-32-1	208-534-8
POTASSIUM SORBATE	0.1	24634-61-5 / 590-00-1	246-376-1

The ingredients and their quantities are shown, as indicated by the producer.

Table S2. Mass spectral data of the identified compounds.

Compound	Formula	z	RT	[M-H]- exp (m/z)	MS2 ions (m/z)
Citric acid	$C_6H_8O_7$	-1	1.15	191.0186	111,0074; 87,0073
Punicalagin isomer	$C_{48}H_{28}O_{30}$	-2	8.32	541.0259	300,9987; 275,0197; 600,9899; 781,0546;
Punicalagin isomer	$C_{48}H_{28}O_{31}$	-2	6.06	541.0259	300,9987; 275,0197; 600,9899; 781,0546;
Brevifolin carboxylix acid	$C_{13}H_8O_8$	-1	8.35	291.0143	247,0296; 191,0347
Punicalagin isomer	$C_{48}H_{28}O_{31}$	-2	2.94	541.0259	300,9987; 275,0197; 600,9899; 781,0546;
Ellagic acid	$C_{14}H_6O_8$	-1	15.13	300.9986	257.01
Galloyl-HHDP-hexoside	$C_{27}H_{22}O_{18}$	-1	10.39	633.0733	300,9990; 275,0197; 463,0509
Gallic acid	$C_7H_7O_5$	-1	1.76	169.0131	125.02
Punicalin	$C_{34}H_{22}O_{22}$	-1	2.03	781.0530	600,9896; 721,0267
HHDP-hexoside isomer	$C_{20}H_{18}O_{14}$	-1	1.10	481.0623	300,9989; 275,0199
HHDP-hexoside isomer	$C_{20}H_{18}O_{14}$	-1	1.26	481.0622	300,9989; 275,0200
Ellagic acid -hexoside	$C_{20}H_{16}O_{13}$	-1	12.03	463.0519	300,9988; 299,9910
Pedunculagin isomer	$C_{34}H_{24}O_{22}$	-1	1.65	783.0692	299,0193; 300,9993; 275,0191
di (HHDP-galloyl-hexoside)-pentoside		-2	4.15	707.0637	300,9989; 275,0195; 613,0460; 783,0692
Galloyl-HHDP-hexoside	$C_{27}H_{22}O_{18}$	-1	1.97	633.0734	300,9990; 275,0197
di (HHDP-galloyl-hexoside)-pentoside		-2	4.51	707.0637	300,9989;<br>613,0460; 275,0195; 633,0740
Galloyl-HHDP-hexoside	$C_{27}H_{22}O_{18}$	-1	2.89	633.0735	300,9990; 275,0197
Pedunculagin isomer	$C_{34}H_{24}O_{22}$	-1	2.52	783.0692	275,0197; 299,0206; 301,0000
Digalloyl-hexoside isomer	$C_{20}H_{20}O_{14}$	-1	8.06	483.0780	169,0132; 125,0232; 331,0669; 313,0567
Digalloyl-hexoside isomer	$C_{20}H_{20}O_{14}$	-1	3.73	483.0782	169,0132; 125,0232; 331,0669; 313,0567
Galloyl-hexoside isomer	$C_{13}H_{16}O_{10}$	-1	1.62	331.0670	271,0461; 169,0131: 125,0230
Ellagic acid-deoxyhexoside	$C_{20}H_{16}O_{12}$	-1	15.35	447.0569	299,9910; 300,9988
Brevifolin	$C_{12}H_8O_6$	-1	11.45	247.0243	191,0341; 219,0301
Galloyl-hexoside isomer	$C_{13}H_{16}O_{10}$	-1	1.92	331.0667	271,0461; 169,0131: 125,0231
Galloyl-hexoside isomer	$C_{13}H_{16}O_{10}$	-1	1.08	331.0670	169,0131: 125,0230; 271,0461; 241,0348
Galloyl-hexoside isomer	$C_{13}H_{16}O_{10}$	-1	1.42	331.0671	169,0131: 125,0230; 271,0461; 241,0349
Pedunculagin isomer	$C_{34}H_{24}O_{22}$	-1	4.77	783.0692	300,9993; 275,0196
Digalloyl-hexoside isomer	$C_{20}H_{20}O_{14}$	-1	2.00	483.0776	169,0132; 125,0232; 331,0669; 313,0567
di (HHDP-galloyl-hexoside)-pentoside		-2	7.71	707.0634	300,9991; 275,0200; 613,0449; 783,0686
Ellagic acid-pentoside	$C_{19}H_{14}O_{12}$	-1	15.07	433.0408	300,9999; 299,9908

For each compound, the chemical formula, the number of negative charges (z), the retention time (RT), the [M-H] - exp (m/z) are shown, as provided by the instrument. Also, the MS2 ions (m/z) corresponding to compound fragmentation profile used to identify the structure of the specific compound are given.

Table S3. Polyphenolic content in PomeGr exposed or not to *C. albicans*.

		**Levels of polyphenolic compounds**		
Compounds	***Peak number***	***PomeGr alone (AUP)***	***PomeGr +C. albicans (AUP)***	***% Decrease***
Galloyl-hexoside isomer 1	1	$7.82x10^{6} \pm 2.71x10^{5}$	$7.52x10^{6} \pm 4.34x10^{5}$	**3.8**
HHDP-hexoside isomer 1	2	$3.23x10^{7} \pm 1.94x10^{6}$	$2.75x10^{7} \pm 3.92x10^{6}$	**14.9**
Citric acid	3	$3.29x10^{9} \pm 1.50x10^{8}$	$3.07x10^{9} \pm 1.77x10^{8}$	**6.7**
HHDP-hexoside isomer 2	4	$8.47x10^{7} \pm 1.98x10^{6}$	$6.69x10^{7} \pm 5.24x10^{6}$	**21.0**
Galloyl-hexoside isomer 2	5	$1.35x10^{7} \pm 2.03x10^{6}$	$1.29x10^{7} \pm 5.24x10^{4}$	**4.4**
Galloyl-hexoside isomer 3	6	$1.02x10^{7} \pm 3.40x10^{5}$	$9.57x10^{6} \pm 5.27x10^{5}$	**6.2**
Pedunculagin isomer 1	7	$7.43x10^{6} \pm 1.61x10^{5}$	$462x10^{6} \pm 3.31x10^{5}$	**37.8**
Gallic acid	8	$5.55x10^{7} \pm 2.61x10^{6}$	$5.21x10^{7} \pm 2.51x10^{5}$	**6.1**
Galloyl-hexoside isomer 4	9	$1.29x10^{7} \pm 3.59x10^{5}$	$1.19x10^{7} \pm 6.12x10^{5}$	**7.8**
Galloyl-HHDP-hexoside isomer 1	10	$1.39x10^{7} \pm 1.68x10^{6}$	$1.30x10^{7} \pm 7.71x10^{5}$	**6.5**
Digalloyl-hexoside isomer 1	11	$1.09x10^{7} \pm 7.88x10^{5}$	$8.99x10^{6} \pm 6.74x10^{5}$	**17.5**
Punicalin	12	$6.48x10^{7} \pm 2.40x10^{6}$	$4.32x10^{7} \pm 2.63x10^{6}$	**33.3**
Pedunculagin isomer 2	13	$9.07x10^{6} \pm 9.81x10^{4}$	$5.32x10^{6} \pm 3.00x10^{5}$	**41.3**
Galloyl-HHDP-hexoside isomer 2	14	$1.25x10^{7} \pm 1.42x10^{6}$	$1.01x10^{7} \pm 5.31x10^{5}$	**19.2**
Punicalagin isomer 1	15	$3.70x10^{7} \pm 1.23x10^{6}$	$2.26x10^{7} \pm 1.17x10^{6}$	**38.9**
Digalloyl-hexoside isomer 2	16	$8.31x10^{6} \pm 8.94x10^{4}$	$7.25x10^{6} \pm 1.99x10^{5}$	**12.8**
Granatin isomer 1	17	$2.44x10^{6} \pm 8.26x10^{4}$	$1.57x10^{6} \pm 6.60x10^{4}$	**35.7**
Di-[hexahydroxydiphenoyl (HHDP)-galloyl-hexoside]-pentoside	18	$5.56x10^{6} \pm 2.70x10^{5}$	$3.56x10^{6} \pm 2.01x10^{5}$	**36.0**
Granatin isomer 2	19	$1.47x10^{6} \pm 7.41x10^{4}$	$8.80x10^{5} \pm 1.18x10^{5}$	**40.1**
Punicalagin isomer 2	20	$3.61x10^{7} \pm 1.02x10^{6}$	$2.04x10^{7} \pm 3.65x10^{5}$	**43.5**
Punicalagin isomer 3	21	$8.04x10^{7} \pm 8.61x10^{5}$	$4.78x10^{7} \pm 1.51x10^{6}$	**40.5**
Brevifolin carboxylic acid	22	$9.55x10^{7} \pm 2.09x10^{6}$	$8.69x10^{7} \pm 2.63x10^{6}$	**9.01**
Galloyl-HHDP-hexoside isomer 3	23	$9.39x10^{7} \pm 2.93x10^{6}$	$7.30x10^{7} \pm 4.98x10^{5}$	**22.3**
Ellagic acid -hexoside	24	$1.93x10^{7} \pm 3.28x10^{5}$	n.d.	**100**
Ellagic acid	25	$4.08x10^{7} \pm 2.64x10^{6}$	$3.75x10^{7} \pm 2.46x10^{4}$	**8.1**
Ellagic acid-deoxyhexoside	26	$5.21x10^{6} \pm 1.51x10^{5}$	$4.28x10^{6} \pm 2.80x10^{5}$	**17.9**

Phenolic compounds were identified by HPLC-ESI-MS in PomeGr alone and after incubation with *C. albicans* (24 h). The relative amounts of each polyphenol were measured by the ion chromatogram, according to the peak area of each compound, and were expressed as AUP arbitrary units (tolerance ± 5 ppm). The % decrease was calculated comparing the AUP of treated vs untreated groups. n.d.: compound not detected
